# Laser-Assisted Self-Monitoring of Blood Glucose: Analytical Performance, Clinical Accuracy, and Usability of the HandyRay-Glu System

**DOI:** 10.3390/diagnostics16111700

**Published:** 2026-05-31

**Authors:** Minsup Lim, JunMin Lee, Ji A Seo, Sun-Young Ko

**Affiliations:** 1 Department of Health and Safety Convergence Science, School of Health Science, Korea University, Seoul 02841, Republic of Korea; limminsup@naver.com; 2 Department of Laboratory Medicine, Korea University Guro Hospital, Korea University College of Medicine, Seoul 02842, Republic of Korea; dlwnsals15@korea.ac.kr; 3 Division of Endocrinology and Metabolism, Department of Internal Medicine, Korea University Ansan Hospital, Korea University College of Medicine, Seoul 02842, Republic of Korea; 4 Department of Laboratory Medicine, Korea University Ansan Hospital, Korea University College of Medicine, Seoul 02842, Republic of Korea

**Keywords:** laser-assisted blood sampling, self-monitoring of blood glucose, blood glucose monitoring system, ISO 15197

## Abstract

**Background/Objectives**: Diabetes mellitus is a major global health burden, and inadequate glycemic control increases the risk of microvascular and macrovascular complications. Self-monitoring of blood glucose (SMBG) is essential for diabetes management, but conventional finger-prick sampling may reduce adherence due to pain and repeated skin injury. This study evaluated the analytical performance, clinical accuracy, and usability of a novel laser-assisted blood glucose monitoring system, HandyRay-Glu. **Methods**: A prospective clinical evaluation study was conducted in accordance with ISO 15197:2013. Capillary blood glucose values obtained using the HandyRay-Glu system were compared with reference measurements generated by the cobas c111 analyzer. Analytical performance was assessed by evaluating repeatability, linearity, hematocrit effect, and interference. Clinical performance was assessed according to ISO 15197:2013 system accuracy criteria, and method comparison was performed using Passing–Bablok regression and Bland–Altman analyses. Usability was evaluated using a structured participant questionnaire. **Results**: A total of 100 adult participants with diabetes mellitus were included. Overall, 97.8% of results met the ISO 15197:2013 accuracy criteria. Passing–Bablok regression showed strong agreement between HandyRay-Glu and the reference method (y = 1.694 + 0.9859x, r = 0.992). Bland–Altman analysis demonstrated a mean bias of −1.763 mg/dL, with 95% limits of agreement ranging from −29.333 to 25.808 mg/dL. Analytical evaluations showed acceptable repeatability, linearity across the tested measurement range, and no clinically significant interference. More than 97% of participants reported satisfaction with device usability. **Conclusions**: The HandyRay-Glu system met the performance requirements of ISO 15197:2013 and demonstrated high analytical accuracy, acceptable agreement with the reference method, and favorable usability. Laser-assisted blood sampling combined with electrochemical glucose measurement may offer a potential alternative to conventional SMBG systems, and its possible role in improving patient acceptance of regular monitoring warrants further investigation.

## 1. Introduction

Diabetes mellitus is one of the most prevalent chronic metabolic disorders worldwide, and its global burden continues to increase [1]. According to the International Diabetes Federation, the number of individuals living with diabetes remains substantial and is expected to rise further in the coming decades. Poor glycemic control is closely associated with the development of both microvascular complications, such as retinopathy, nephropathy, and neuropathy, and macrovascular complications, including cardiovascular disease. These complications contribute substantially to morbidity, mortality, reduced quality of life, and healthcare expenditure, emphasizing the importance of effective glucose monitoring and glycemic management in routine clinical practice.

Self-monitoring of blood glucose (SMBG) plays a critical role in diabetes management by enabling patients to assess glycemic variability in daily life. SMBG provides information that cannot be captured adequately by intermittent hospital-based testing alone, particularly because glucose levels vary dynamically throughout the day. Evidence from clinical studies has demonstrated that SMBG contributes to improved glycemic control, especially when the results are actively used to guide behavioral or therapeutic adjustments [2,3,4]. Accordingly, SMBG has become an integral component of diabetes self-management and an important source of information for clinicians managing antidiabetic treatment [5].

Despite its clinical utility, conventional SMBG systems rely on repeated finger-prick sampling using mechanical lancets, which is associated with pain, local skin injury, and psychological discomfort [6]. These factors can negatively affect adherence to regular glucose monitoring, particularly in individuals requiring frequent or long-term testing, such as those receiving insulin therapy or those at increased risk of hypoglycemia. Accordingly, there is growing interest in alternative sampling technologies that reduce patient burden while maintaining the analytical accuracy necessary for clinical decision-making.

Laser-assisted sampling has emerged as one such alternative approach. By creating a controlled micro-perforation in the skin, laser-based systems may enable capillary blood collection with reduced pain and less mechanical trauma than conventional lancets [7]. The HandyRay-Glu personal blood glucose monitoring system combines laser-assisted capillary blood sampling with electrochemical glucose measurement in a single integrated device. The system employs an Er:YAG laser to obtain a small capillary sample and uses a glucose dehydrogenase-flavin adenine dinucleotide (GDH-FAD)-based electrochemical method for glucose detection. Because blood glucose monitoring systems intended for self-testing must satisfy internationally accepted accuracy requirements, performance evaluation according to ISO 15197:2013 is essential for demonstrating clinical utility [8]. In this study, we evaluated the analytical performance, clinical accuracy, and usability of the HandyRay-Glu system in accordance with ISO 15197:2013.

## 2. Materials and Methods

### 2.1. Study Design and Participants

This prospective clinical evaluation study was conducted at Korea University Ansan Hospital between 27 August and 29 August 2025. The study was designed to evaluate the analytical and clinical performance of the HandyRay-Glu blood glucose monitoring system in accordance with ISO 15197:2013, which specifies minimum requirements for blood glucose monitoring systems intended for self-testing in the management of diabetes mellitus. The protocol was reviewed and approved by the Institutional Review Board of Korea University Ansan Hospital (IRB No. 2025AS0128, approved on 10 June 2025), and the study was conducted in accordance with the Declaration of Helsinki and Good Clinical Practice guidelines. Written informed consent was obtained from all participants before enrollment and study-related procedures.

Adult patients aged 19 to 80 years with physician-diagnosed diabetes mellitus who were receiving outpatient care were invited to participate. Eligibility criteria were established with reference to ISO 15197:2013 [8]. Participants were required to be capable of providing fingertip capillary blood samples and of performing self-monitoring blood glucose procedures using a personal blood glucose monitoring system. Only participants who voluntarily agreed to participate and provided written informed consent after receiving an explanation of the study objectives, procedures, and possible risks were enrolled.

Participants were excluded if they had hematological abnormalities that could influence glucose measurement, such as severe anemia or polycythemia; unstable systemic conditions, including acute infection, severe hepatic dysfunction, or renal failure; skin disorders, infection, or injury at the sampling site that could interfere with safe blood collection; pregnancy or breastfeeding; or any other condition that the investigator considered inappropriate for study participation. In addition, enrollment was performed with consideration of the glucose concentration and hematocrit distribution required under ISO 15197:2013 so that the study population would be appropriate for the evaluation of system accuracy across clinically relevant ranges.

A total of 100 participants were included in the final analysis. The target sample size was determined based on the user performance requirements of ISO 15197:2013 [8], which recommends the inclusion of at least 100 intended users and a minimum of 200 paired glucose measurements for system accuracy evaluation under lay-user conditions.

### 2.2. Test Device and Reference Analyzer

The investigational device was the HandyRay-Glu Blood Glucose Test Meter (BL-6000) (Lameditech, Seoul, Republic of Korea) used with HandyRay-Glu Blood Glucose Test Strips (OG-SH21-GHS) (Osang Healthcare, Anyang, Republic of Korea). The system integrates laser-assisted blood sampling and electrochemical glucose analysis in a single device. The reference method for comparison was the cobas c111 analyzer (Roche Diagnostics, Basel, Switzerland), which was operated according to the manufacturer’s instructions. Before reference testing, calibration and quality control procedures were performed in accordance with routine laboratory protocols, including the use of internal quality control materials to confirm acceptable analytical performance.

### 2.3. Measurement Procedure

Reference blood glucose measurements were performed using the cobas c111 analyzer according to the manufacturer’s instructions. Fresh capillary blood samples obtained within 5 min after measurement with the HandyRay-Glu system were used for the reference analysis. Each sample was measured at least twice using the reference analyzer, and the mean of the replicate measurements was used as the reference glucose value for comparison. Capillary blood samples were analyzed promptly, and measurements obtained from the cobas c111 analyzer were considered plasma-equivalent values in accordance with standard glucose monitoring system evaluation practices, consistent with a point-of-care comparison protocol under the ISO 15197:2013 framework.

Participants performed a single SMBG measurement using the HandyRay-Glu system under simulated lay-user conditions. They were provided with the user manual and accompanying instructional materials but did not receive additional professional guidance during device operation. Before testing, participants prepared the device by checking its charging status, attaching a disposable cap to the laser emission port, and washing and drying their hands. After the device was powered on, the laser intensity was adjusted according to the user’s skin thickness, and a glucose test strip was inserted to activate measurement mode.

The laser emission port was placed firmly against the lateral side of the fingertip to obtain a capillary blood sample. If necessary, a gentle massage of the fingertip was permitted to facilitate blood flow. The resulting blood sample was then applied to the test strip, and the glucose value displayed by the device was recorded. After the measurement, the disposable cap was discarded, and the laser lens area was disinfected with an alcohol swab according to the device procedure. Measurement data were also transmitted to a mobile application via Bluetooth. Measurement errors were predefined as device-generated error messages (e.g., insufficient sample volume, improper strip insertion, or device malfunction) or failure to obtain a valid numerical glucose result. Only such technically invalid measurements were excluded from analysis, and repeat measurements were allowed in these cases, with up to three exclusions permitted per participant. No selective removal of outliers was performed, and this approach is consistent with real-world SMBG use and the ISO 15197 evaluation framework.

### 2.4. Analytical Performance Evaluation

Analytical performance was evaluated in accordance with ISO 15197:2013. The evaluation comprised repeatability, hematocrit effect, interference testing, and linearity assessment.

Repeatability was assessed using three test strip lots across five glucose concentration ranges. For glucose concentrations below 100 mg/dL, repeatability was evaluated using standard deviation (SD), whereas for concentrations of 100 mg/dL or greater, the coefficient of variation (CV) was used in accordance with ISO 15197:2013 criteria.

The effect of hematocrit was evaluated across six hematocrit levels (18%, 26%, 34%, 42%, 50%, and 66%), with 42% used as the reference level. Measurements were performed across low, medium, and high glucose ranges, and the percent bias relative to the reference hematocrit condition was calculated.

Interference testing was performed using 24 potentially interfering substances, including acetaminophen, at concentrations recommended by ISO 15197:2013. For glucose concentrations below 100 mg/dL, bias was evaluated in mg/dL, whereas for concentrations of 100 mg/dL or greater, bias was evaluated in percentage terms.

Linearity was assessed across 11 stepwise glucose concentration levels covering the measurement range of 10–600 mg/dL. Mixed blood samples spanning the target range were analyzed using both the reference analyzer and the HandyRay-Glu system. For concentrations outside the reportable range, device outputs (“Low” and “High”) were recorded.

### 2.5. Clinical Performance Evaluation

Clinical performance was evaluated according to the system accuracy requirements of ISO 15197:2013. For glucose concentrations below 100 mg/dL, HandyRay-Glu results were evaluated based on whether they fell within ±15 mg/dL of the reference method. For concentrations of 100 mg/dL or greater, results were evaluated based on whether they fell within ±15% of the reference method. The overall proportion of results meeting these criteria was calculated and compared with the ISO 15197:2013 minimum acceptance requirement of 95%.

Method comparison between HandyRay-Glu and the reference analyzer was assessed using Passing–Bablok regression and Bland–Altman analysis. Passing–Bablok regression was applied as a nonparametric method for method comparison, without assuming a normal distribution of measurement errors. Bland–Altman analysis was used to assess agreement and bias between the two methods across the measurement range. Absolute difference and relative difference analyses were both considered.

Clarke Error Grid analysis was additionally performed using paired glucose values obtained from the HandyRay-Glu system and the reference analyzer. Each paired measurement was categorized into zones A–E according to standard Clarke Error Grid criteria to assess the clinical significance of measurement discrepancies [9].

### 2.6. Usability Assessment

Usability was evaluated using a structured questionnaire completed by participants after the use of the HandyRay-Glu device. The questionnaire assessed the ease of following the user manual, the readability of the displayed test results, and overall satisfaction with the device and operating procedure. Each item was scored on a 5-point Likert scale (1 = strongly disagree to 5 = strongly agree). Responses were summarized descriptively.

### 2.7. Statistical Analysis

Passing–Bablok regression analysis was performed to evaluate the relationship between HandyRay-Glu and the reference analyzer. Regression slope and intercept were estimated, and the correlation between methods was described using Pearson’s correlation coefficient. Bland–Altman analysis was used to calculate mean bias and 95% limits of agreement for both absolute and relative differences. Analytical performance and ISO system accuracy results were summarized using descriptive statistics. Passing–Bablok regression and Bland–Altman analyses were performed using MedCalc version 22.016 (MedCalc Software Ltd., Ostend, Belgium). Descriptive statistics were analyzed using SPSS version 27.0 (IBM Corp., Armonk, NY, USA).

### 2.8. Use of GenAI Tools

During the preparation of this manuscript, the authors used ChatGPT version 5.4 (OpenAI, San Francisco, CA, USA; accessed via ChatGPT Plus subscription, March 2026) for manuscript organization and English-language editing. The tool was not used for study design, data collection, analysis, or interpretation. The authors have reviewed and edited the output and take full responsibility for the content of this publication.

## 3. Results

### 3.1. Analytical Performance

#### 3.1.1. Repeatability

Measurement repeatability was evaluated using three test strip lots across five glucose concentration ranges. For glucose concentrations < 100 mg/dL, the standard deviation (SD) did not exceed 5 mg/dL, and for concentrations ≥ 100 mg/dL, the coefficient of variation (CV) did not exceed 5%. All results met the acceptance criteria specified in ISO 15197:2013. Detailed results are presented in Table 1.

#### 3.1.2. Hematocrit Evaluation

The effect of hematocrit (Hct) was evaluated at six levels (18%, 26%, 34%, 42%, 50%, and 66%), with 42% used as the reference level. Glucose concentrations were tested across low (30–50 mg/dL), medium (96–144 mg/dL), and high (280–420 mg/dL) ranges. Across all concentration ranges, the percent bias relative to the reference condition did not exceed 10%, and all measurements met the predefined acceptance criteria.

#### 3.1.3. Interference Testing

A total of 24 potential interfering substances, including acetaminophen, were evaluated at selected concentrations. For glucose concentrations < 100 mg/dL, the mean bias did not exceed 10 mg/dL, while for glucose concentrations ≥ 100 mg/dL, the mean bias did not exceed 10%. No clinically significant interference was observed.

#### 3.1.4. Linearity

**Figure 1:**
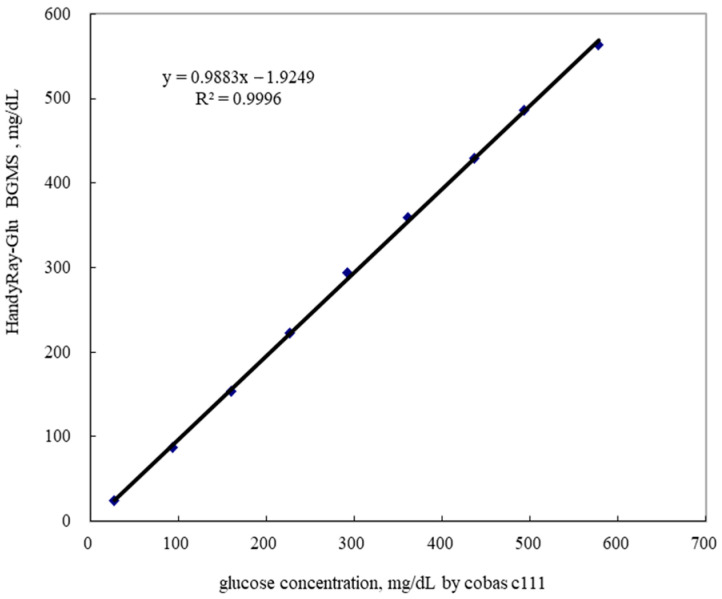
X–Y linearity plot comparing glucose measurements obtained using the HandyRay-Glu system and the cobas c111 analyzer. The regression equation was y = 0.9883x − 1.9249 (R^2^ = 0.9996).

Linearity was evaluated across 11 stepwise glucose concentration levels spanning approximately 10–600 mg/dL. The device displayed “Low” for concentrations below 10 mg/dL and “High” for concentrations above 600 mg/dL. Within the measurable range, regression analysis demonstrated excellent linearity between the test device and the reference analyzer (R^2^ = 0.9996; y = 0.9883x − 1.9249). The linearity results are shown in Figure 1.

### 3.2. Clinical Performance

#### 3.2.1. User Accuracy

Clinical accuracy under user-operated conditions was evaluated in 100 adult participants with diabetes mellitus. For glucose concentrations below 100 mg/dL, 98% of measurements met the ISO 15197:2013 criterion of being within ±15 mg/dL of the reference value. For glucose concentrations of 100 mg/dL or greater, 97% of measurements met the criterion of being within ±15% of the reference value. Overall, 97.8% of all paired results met the ISO 15197:2013 system accuracy requirement. Among the total measurements, 12 (12.0%) were within the hypoglycemic range (<70 mg/dL), 38 (38.0%) were between 70 and 100 mg/dL, and 50 (50.0%) were ≥100 mg/dL. In the hypoglycemic range, 11 of the 12 measurements (91.7%) met the ISO 15197:2013 accuracy criteria. The distribution of glucose values across clinically relevant ranges is summarized in Table 2.

#### 3.2.2. Method Comparison

**Figure 2:**
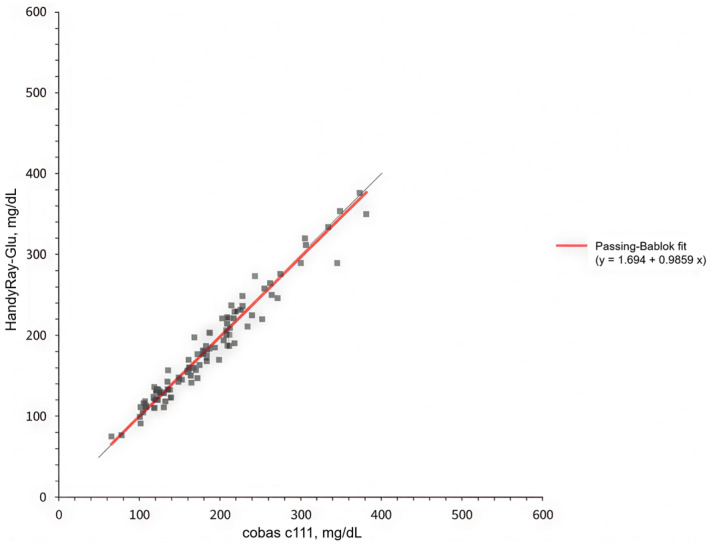
Passing–Bablok regression plot comparing glucose measurements obtained using the HandyRay-Glu system and the cobas c111 analyzer. Glucose concentrations measured by cobas c111 (*x*-axis) and HandyRay-Glu (*y*-axis) are shown. Each square represents an individual sample. The red line indicates the Passing–Bablok regression fit (y = 1.694 + 0.9859x), and the gray line represents the line of identity (y = x).

Passing–Bablok regression analysis showed a regression equation of y = 1.694 + 0.9859x (Figure 2). Pearson correlation analysis demonstrated a strong linear association between the two methods (r = 0.992; 95% CI, 0.988–0.995; *p* < 0.001), with a coefficient of determination of R^2^ = 0.984.

**Figure 3:**
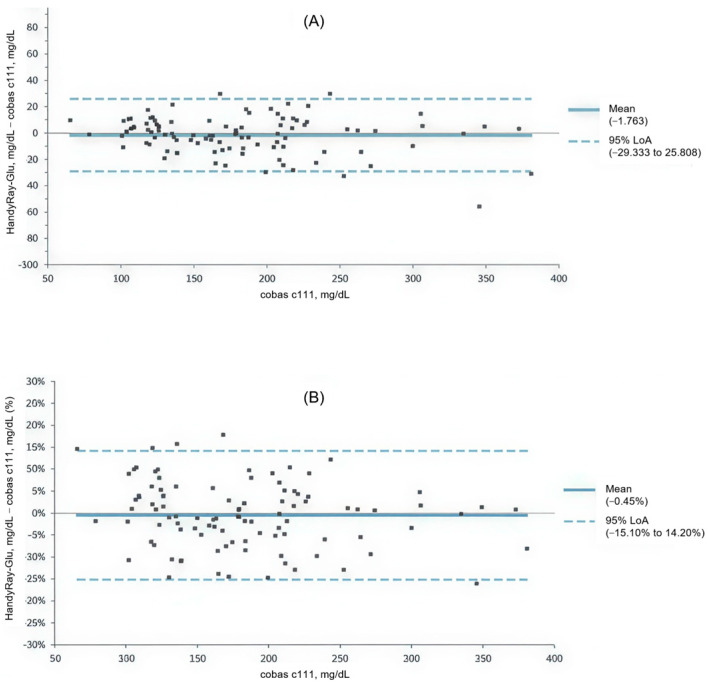
Bland–Altman plots comparing glucose measurements obtained using the HandyRay-Glu system and the cobas c111 analyzer. (**A**) Absolute difference plot. The difference in glucose concentrations (HandyRay-Glu − cobas c111, mg/dL) is plotted against the glucose concentration measured by cobas c111. The solid line represents the mean difference (−1.763 mg/dL), and the dashed lines indicate the 95% limits of agreement (−29.333 to 25.808 mg/dL). (**B**) Relative difference plot. The percentage difference (HandyRay-Glu − cobas c111, %) is plotted against the glucose concentration measured by cobas c111. The solid line represents the mean difference (−0.45%), and the dashed lines indicate the 95% limits of agreement (−15.10% to 14.20%).

Bland–Altman analysis showed a mean bias of −1.763 mg/dL, with 95% limits of agreement ranging from −29.333 to 25.808 mg/dL (Figure 3). In the relative difference analysis, the mean bias was −0.45%, with 95% limits of agreement ranging from −15.10% to 14.20%. Most paired measurements were distributed within the 95% limits of agreement, and no obvious trend in bias was observed across the measurement range.

#### 3.2.3. Clarke Error Grid Analysis

The Clarke Error Grid analysis showed that 96% of results were in zone A, and 4% were in zone B. No results were observed in zones C–E.

### 3.3. Usability Assessment

Among the participants, 96.0% reported prior experience using a blood glucose meter, and 63.0% had more than four years of experience. Regarding usability, 99.0% of participants reported that the instructions in the user manual were easy to follow, and 99.0% reported that the test results displayed on the meter were easy to read. Overall, 97.0% of participants reported satisfaction with the user manual and device operation (score ≥ 3 on a 5-point scale). Detailed questionnaire results are presented in Table 3.

## 4. Discussion

ISO 15197:2013 defines the minimum analytical and user performance requirements for blood glucose monitoring systems intended for self-testing by people with diabetes [8]. Under this standard, at least 95% of measured values must fall within ±15 mg/dL of the reference method at glucose concentrations below 100 mg/dL or within ±15% at concentrations of 100 mg/dL or greater. In the present study, the HandyRay-Glu system achieved an overall compliance rate of 97.8%, thereby exceeding the minimum ISO requirement. These findings indicate that the system provides analytical and clinical performance consistent with internationally accepted standards for SMBG systems [10,11,12].

The method comparison results also support the clinical reliability of the device [13,14]. Passing–Bablok regression showed close agreement between HandyRay-Glu and the cobas c111 analyzer, with a slope near 1 and a relatively small intercept. Bland–Altman analysis demonstrated a small mean bias and acceptable limits of agreement across the tested concentration range. Taken together, these results suggest that the HandyRay-Glu system yields glucose values closely aligned with those of the reference method, without evidence of clinically meaningful systematic discrepancy. In addition, the Clarke Error Grid analysis indicated that the majority of values fell within clinically acceptable zones, supporting the conclusion that measurement differences are unlikely to lead to clinically significant errors in patient management [9].

In addition to clinical accuracy, the device showed acceptable analytical performance in repeatability, linearity, hematocrit evaluation, and interference testing [10,11,15]. Repeatability remained within ISO acceptance criteria across all tested glucose ranges, indicating stable performance under repeated testing conditions. Linearity demonstrated excellent agreement with the reference analyzer across a broad range of glucose concentrations, supporting a consistent analytical response across clinically relevant levels. The hematocrit study showed that variations within the tested range did not produce clinically unacceptable bias, which is important given the known susceptibility of electrochemical systems to hematocrit-related effects. Similarly, no clinically significant interference was observed among the tested substances, supporting the robustness of the measurement system under common conditions.

The electrochemical detection system used in HandyRay-Glu is based on GDH-FAD, which offers several practical advantages for glucose measurement. Compared with certain alternative enzyme systems, GDH-FAD provides stable performance and is relatively less influenced by oxygen tension [16,17]. Although the present study was not designed as a direct comparison of enzyme chemistries, the favorable analytical and interference results observed here are consistent with the expected advantages of the GDH-FAD-based measurement principle.

A key distinguishing feature of the HandyRay-Glu system is its laser-assisted blood sampling mechanism. One of the main barriers to regular SMBG is the burden imposed by repeated lancet-based sampling, which can lead to pain, local tissue injury, and reduced willingness for repeated testing [6]. The use of an Er:YAG laser enables controlled micro-perforation of the skin, which has been suggested to potentially reduce mechanical trauma compared with conventional lancet-based sampling [7]. In a previous comparative study using the laser sampling component of this platform, capillary blood sampling with the laser device showed strong agreement with conventional lancet-based sampling in glucose values and was associated with significant reductions in puncture pain and improvements in patient satisfaction in patients with diabetes [18]. Although the favorable usability results observed in this study are consistent with the hypothesis that reducing sampling burden may improve user acceptance and adherence to regular glucose monitoring [19,20,21,22], it should be emphasized that the present study did not directly assess pain, local tissue effects, or longitudinal adherence; therefore, these potential benefits remain to be confirmed in dedicated comparative studies.

A theoretical concern with laser-assisted sampling is whether local thermal effects might alter the biochemical properties of the sampled blood and thereby influence glucose measurement. However, the Er:YAG laser operates at a wavelength with high absorption in water, resulting in highly localized superficial tissue ablation with minimal thermal spread [7]. Under these conditions, the collected sample is expected to reflect capillary blood without clinically meaningful alteration. The close agreement observed between HandyRay-Glu and the reference analyzer further supports that the laser-assisted sampling process did not introduce measurable systematic bias under the tested conditions [13,14].

The usability findings also deserve emphasis. Nearly all participants reported that the instructions were easy to follow and that the displayed test results were easy to read, and most participants expressed overall satisfaction with the device. Because the study was conducted under simulated lay-user conditions, these findings are relevant to real-world self-testing use [5,19]. However, the relatively high proportion of participants with prior SMBG experience may have contributed to the favorable usability outcomes, and further evaluation in more diverse user populations is warranted.

Several limitations should be acknowledged. First, this was a single-center study conducted over a relatively short period, which may limit generalizability. Second, the relatively small number of samples within the hypoglycemic range (<70 mg/dL) and very high glucose levels may constrain the reliability of accuracy assessment in these clinically critical regions; accordingly, results within these ranges should be interpreted with caution and not over-interpreted. Third, the study did not include a comparator arm using conventional lancet-based sampling, and objective assessment of pain or local tissue effects was not performed; therefore, the potential advantages of laser-assisted sampling in terms of reduced pain and improved patient acceptance could not be directly substantiated by the present data. Fourth, although usability was assessed using a structured questionnaire, a more detailed evaluation of user errors and device handling would further strengthen the analysis, and longitudinal data on adherence and testing frequency over time were not collected. Fifth, an independent local validation of the cobas c111 analyzer for capillary whole blood samples was not performed in the present study; however, this approach is commonly employed within the ISO 15197-based framework for evaluating blood glucose monitoring systems.

Despite these limitations, the present study demonstrates that the HandyRay-Glu system meets ISO 15197:2013 performance requirements while also showing favorable usability. These findings suggest that the integration of laser-assisted sampling with electrochemical glucose measurement may represent a potential alternative to conventional SMBG systems, although definitive clinical superiority over conventional methods has not been established by the present data. Further studies are warranted, including multi-center investigations across more diverse patient populations; head-to-head comparisons with conventional lancet-based sampling; analyses using validated objective pain assessment tools; longitudinal evaluations of usability, patient compliance, adherence, and testing frequency; and dedicated investigations targeting patients at higher risk of hypoglycemia to substantiate the suggested patient-centered benefits of laser-assisted sampling and to confirm device performance in clinically critical glucose ranges.

## Figures and Tables

**Table 1:** Repeatability of the HandyRay-Glu System across glucose concentration ranges.

No.	Glucose Range (mg/dL)	HandyRay-Glu		
		Mean (mg/dL)	SD (mg/dL)	CV (%)
1	30–50	39.3	1.9	4.8
2	51–110	95.1	3	3.2
3	111–150	150.8	3.4	2.3
4	151–250	226	5.3	2.3
5	251–400	345.9	8.5	2.5

Results were pooled across three test strip lots. Each value represents the mean of replicate measurements across all lots. SD, standard deviation; CV, coefficient of variation.

**Table 2:** Distribution of glucose concentrations and accuracy by range.

Glucose Range (mg/dL)	Number of Samples (*n*)	Percentage (%)	ISO Criteria Met (*n*, %)
<70	12	12.0	11 (91.7%)
70–100	38	38.0	37 (97.4%)
≥100	50	50.0	49 (98.0%)
Total	100	100	97.8% overall

The distribution of glucose values across clinically relevant ranges is summarized. ISO Criteria Met indicates the number and percentage of measurements meeting the ISO 15197:2013 system accuracy criteria within each range.

**Table 3:** User manual and device satisfaction (participants: n = 100).

Description	Strongly Agree	Agree	Neutral	Disagree	Strongly Disagree
The instructions provided in the user manual were easy to follow.	63 (63.0%)	24 (24.0%)	12 (12.0%)	1 (1.0%)	0 (0.0%)
Favorable responses: 99 (99.0%)					
The test results displayed on the device were easy to check.	79 (79.0%)	18 (18.0%)	2 (2.0%)	1 (1.0%)	0 (0.0%)
Favorable responses: 99 (99.0%)					
Overall, I am satisfied with the user manual and device operation.	69 (69.0%)	20 (20.0%)	8 (8.0%)	3 (3.0%)	0 (0.0%)
Favorable responses: 97 (97.0%)					

Data are presented as n (%). Each item was scored on a 5-point Likert scale ranging from 1 (strongly disagree) to 5 (strongly agree). Responses ≥ 3 (neutral or higher) were considered favorable.

## Data Availability

The data presented in this study are available upon request from the corresponding author. The data are not publicly available due to patient privacy restrictions.

## Abbreviations

The following abbreviations are used in this manuscript:

SMBG	Self-monitoring of blood glucose
GDH-FAD	Glucose dehydrogenase-flavin adenine dinucleotide
SD	Standard deviation
CV	Coefficient of variation
Hct	Hematocrit
